# High-power Holmium:Yag lithotripsy in bladder urolithiasis: Is it safe and effective? A combined clinical and experimental study

**DOI:** 10.1080/20905998.2024.2304516

**Published:** 2024-01-21

**Authors:** Konstantinos Pagonis, Paraskevi Katsakiori, Angelis Peteinaris, Vasileios Tatanis, Arman Tsaturyan, Gabriel Faria Costa, Solon Faitatziadis, Athanasios Vagionis, Anastasios Natsos, Kristiana Gkeka, Mohammed Obaidat, Theodoros Spinos, Theofanis Vrettos, Evangelos Liatsikos, Panagiotis Kallidonis

**Affiliations:** a Department of Urology, University of Patras, Patras, Greece; b Department of Urology, Erebouni Medical Center, Yerevan, Armenia; c Department of Urology, Unidade Local de Saúde de Matosinhos, Matosinhos, Portugal; d Department of Surgery and Physiology, Faculty of Medicine of the University of Porto, Porto, Portugal; e Department of Anesthesiology and ICU, University of Patras, Patras, Greece; f Department of Urology, Medical University of Vienna, Vienna, Austria

**Keywords:** High-power laser, bladder lithotripsy, Ho:YAG laser

## Abstract

**Objective:**

To evaluate the efficacy and safety of Holmium: Yttrium-Aluminum-Garnet (Ho:YAG) laser in bladder lithotripsy using high-power settings > 100 W.

**Materials and Methods:**

A combined experimental and clinical study was conducted. The Quanta Cyber: Ho 150 with a 550 μm Quanta optical fiber was utilized in all set-ups. Ablation rates for soft and hard artificial stones were tested in vitro using 100 W and 20 W power settings. In the experiment, a porcine bladder was used. The optical fiber was inserted through a rigid cystoscope, whilst a K-type thermocouple was inserted in the bladder dome. The tested high-power settings were 152 W, 120 W and 105 W. In every trial, the lasing time was over 60 s. In the clinical study, 35 patients underwent transurethral high-power bladder lithotripsy. Laser settings were set between 100 W and 150 W.

**Results:**

Stone mass (stone weight) was significantly lower after stone ablation independently of the stone type or the laser settings. Significantly higher mass decrease and ablation rate were detected in high-power compared to low-power settings. In the experiment, the highest temperature recorded was 32°C at 152 W. At 120 W and 105 W, the peak temperatures didn’t reach 30°C. In the clinical study, a stone-free rate of 100% and a mean operative time of 43 ± 18 min were reported. All patients stayed in the hospital for one day except for one who presented minor hematuria. Additional complications did not occur.

**Conclusion:**

Ho:YAG laser lithotripsy > 100 W is an effective, fast and safe modality for the treatment of bladder calculi.

## Introduction

Urolithiasis has affected mankind since ancient times. The presence of stones in the human bladder, named as bladder lithiasis (BL), represents a small percentage (5%) of the urinary stone disease. BL is usually associated with bladder outlet obstruction (benign prostatic hyperplasia, bladder neck stenosis, urethral strictures, etc.), neurogenic bladder, lower urinary tract infections, surgical complications and rarely with the presence of foreign bodies in the bladder [1]. BL can be managed by both conservative and surgical methods. Surgical treatment includes open cystolithotomy and litholapaxy, extracorporeal shock wave percutaneous and transurethral pneumatic and laser lithotripsy. In addition, combined techniques including transurethral and percutaneous approaches have also been described [2].

The use of lasers in the treatment of urolithiasis has evolved significantly over time. In combination with the miniaturization of the endoscopic equipment, the transition from low-power (LP) to high-power (HP) lithotripsy constituted a milestone that affected the operative outcomes [3]. The use of HP lithotripsy has been associated with improved operative time, while the dusting has constituted a widely used technique [4,5]. Currently, Holmium: Yttrium-Aluminum-Garnet (Ho:YAG), which can provide HP settings, is widely utilized in the performance of endoscopic surgeries in urology [6].

Laser lithotripsy is considered an effective, minimally invasive and fast procedure for the management of BL as it is associated with improved stone removal and shorter hospitalization [7]. Moreover, it seems to be a feasible alternative in the pediatric population, in which the presence of bladder stones is mainly associated with metabolic or genetic factors [8]. However, controversy exists regarding the power settings that should be used for safe, fast, and effective bladder lithotripsy. In search of optimal lithotripsy parameters, several studies have been published that evaluate the temperature increase and the ablation rate (AR) of various HP holmium laser bladder lithotripsy settings. In their clinical trial, Bhat *et al*. reported clear superiority of HP bladder lithotripsy with 100 W, as it demonstrated reduced operative time without affecting the complication rate [9]. In contrast, HP settings have been described to increase irrigation fluid temperatures and carry potentially harmful effect on the bladder [10].

The present study aims to investigate the safety and efficacy of Ho:YAG laser bladder lithotripsy using HP settings > 100 W through experimental in-vitro and ex-vivo as well as observational clinical study.

## Materials and methods

Three different settings were utilized in this study. The in-vitro setting was used for the estimation of the stone mass decrease as well as the stone AR based on the type of the stone and the utilized power settings. The increase of the irrigation fluid temperature associated with various power settings was evaluated in the ex-vivo setting. Finally, a prospective clinical observation study was conducted to assess the safety and clinical outcome of HP bladder lithotripsy. In all settings, the Quanta Cyber: Ho 150 (Quanta System, Samarate, Italy) with the 550 μm Quanta optical fiber (Quanta System, Samarate, Italy) was utilized.

### In-vitro experimental setting

For the purposes of the in-vitro setting, artificial stones were formed. Six hard and six soft stones were prepared in the same 8.5 × 8.5 × 5.5 mm mold with a flat surface [11]. BegoStone^TM^ powder (BegoStone Plus, Bremen, Germany) was mixed with water in a ratio of 15:3 and 15:6 for the creation of the artificial hard and soft stones, respectively [12,13]. The stones were placed in a container filled with saline. Prior to the experimental trial, the stones were soaked for 30 min. A plastic container (45 × 35 × 25 cm) with normal saline and a 10:01 crystal tube fixed to an iron holder were additionally used.

The 550 μm laser fiber was inserted through an 8 Fr‐84 cm guide ureteral catheter (Cook Medical, Bloomington, Indiana) which was advanced through the 16 Fr‐30 cm Amplatz renal dilator (Cook Medical, Bloomington, Indiana). To fix the stones and eliminate the possibility of retropulsion, an 18 Fr‐30 cm Amplatz renal dilator (Cook Medical, Bloomington, Indiana) was inserted through the opposite side of the crystal tube and firmly attached to the stone. Two power settings, HP (100W = 2Jx50Hz) and LP (20W = 0.5Jx40Hz), were tested. In each trial, lithotripsy was performed until it reached a total energy amount of 3 kJ. The generated stone fragments were labeled and left to dry at room temperature for 72 hours. Stone masses were calculated prior to and 72 hours post-lithotripsy. The stone AR was defined as the stone mass difference prior to and 72 hours post lithotripsy over the time needed to reach a total energy amount of 3 kJ.

### Ex-vivo experimental setting

**Figure 1.**
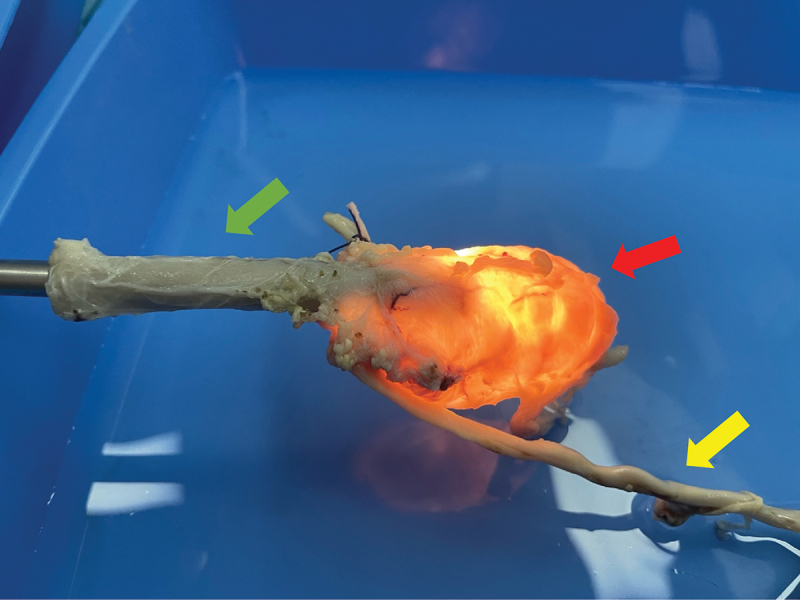
Experimental ex vivo setting.

**Figure 2.**
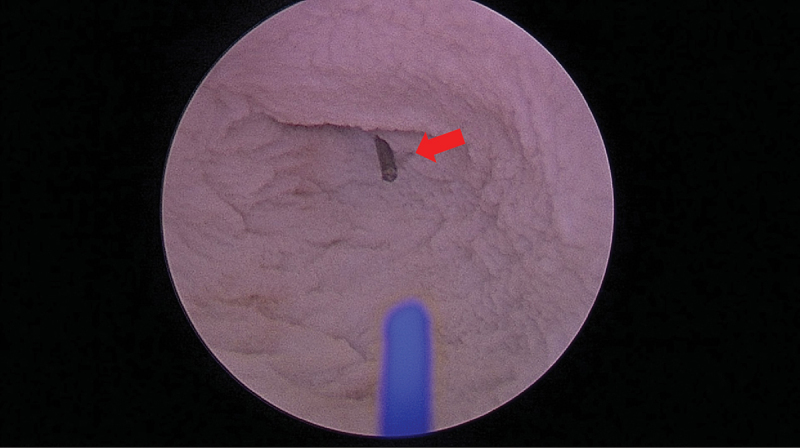
Live tracking of temperature changes via a type K thermocouple in the bladder dome during the ex vivo trials.

A bladder specimen was obtained from a sacrificed pig. The study was conducted after the approval of the veterinary and ethical committee. Afterwards, it was retained in formalin for 14 days. To estimate the irrigation fluid temperature, the bladder was transferred into a container full of saline and cystoscopy using a 22 Fr cystoscope (Storz, Tuttlingen, Germany) was performed (Figure 1). A K-type thermocouple (Pico Technologies, Cambridgeshire, UK) was inserted in the bladder dome for live tracking of the temperature changes (Figure 2). Thereafter, the 550 μm laser fiber was inserted and the laser was activated continuously for 60 s. For irrigation purposes, two bags containing 3 L normal saline each were used. The bags were set 1 meter above the experimental table with continuous irrigation flow. Three HP settings (105W = 1.5Jx70Hz, 120W = 2.4Jx50Hz and 152W = 3.8Jx40hZ) were evaluated.

Red Arrow: Porcine Bladder

Green Arrow: Cystoscope inserted into the porcine urethra

Yellow Arrow: Porcine Ureter

Red Arrow: Type K thermocouple

### Clinical study

A prospective clinical investigation was conducted. The study was approved by the Institutional Ethics Board and has been performed in accordance with the principles stated in the Declaration of Helsinki. Informed consent was obtained from all the patients prior to their participation in the study. All patients who underwent HP bladder lithotripsy in our Urology Department between August 2022 and March 2023 were eligible to participate in the study. Patients with bladder anatomical disorders, urethral strictures and uncontrolled bleeding disorders were excluded. The age, gender and Body Mass Index (BMI, Kgr/m^2^) of the patients, the number and size of the bladder stones, the operative time, the stone clearance, the length of hospital stay, and the presence of complications (based on the Clavien-Dindo classification) were recorded. All procedures were performed under general anesthesia after the anesthesiologist’s recommendations. The laser power settings varied between 100 W and 150 W.

### Statistical analysis

In the experimental part, paired t-test was used for the comparison of the stone mass prior to and post lithotripsy. Unpaired t-test was used to compare the differences in both mass and AR decrease regarding the power settings used. The t-statistic value (the degrees of freedom) and the significance value of the test are presented as t(df) and p, respectively. The confidence interval was set at 95%. Continuous variables are presented as mean±standard deviation whereas categorical variables are shown in percentages. In all statistical analyses, the threshold of statistical significance was set at a two-tailed p-value of 0.05.

## Results

### In vitro evaluation of stone ablation

#### Mass decrease

The pre- and post-lithotripsy mean mass of hard stones was 344 ± 25.68 mgr and 307.5 ± 22.17 mgr, respectively. The mean mass of soft stones was 222.67 ± 15.55 mgr before and 148.83 ± 15.51 mgr after lithotripsy. The reduction in stone mass reached the statistically significant level independently of the laser settings (t [5] = 5.69, *p* = 0.0023 in hard stones and t [5] = 10.37, *p* = 0.0001 in soft stones). Evaluating the HP and LP subgroups, significantly reduced stone mass was detected post lithotripsy in both of them (t [5] = 7.82, *p* = 0.0005 and t [5] = 5, *p* = 0.0041, respectively). Additionally, the comparison between HP and LP favored the HP settings in terms of mass decrease (t [10] = 2.44, *p* = 0.0352).

#### Ablation rate

The comparison of AR between the hard and soft stones did not detect any significant differences (t [10] = 1.59, *p* = 0.1436). Nevertheless, in terms of power settings, LP was associated with significantly lower AR compared to HP, independently of the stone type (t [4] = 13.34, *p* = 0.0002 in hard stones and t [4] = 19.61, *p* < 0.0001 in soft stones). The results of the in vitro experimental study are summarized in Table 1.

**Table 1.** In vitro experimental study of stone ablation.

Stone Hardness	Power (W)	Energy (J)	Frequency (Hz)	Initial Stone Mass (mgr)	Final Stone Mass (mgr)	Time until 3 KJ (s)	Calculated ablation rate (mgr/s)
Hard	100	2	50	352	301	300	0.17
Hard	100	2	50	343	298	300	0.15
Hard	100	2	50	367	312	300	0.183
Hard	20	0.5	40	374	350	700	0.034
Hard	20	0.5	40	313	295	700	0.026
Hard	20	0.5	40	315	289	700	0.037
Soft	100	2	50	248	153	300	0.317
Soft	100	2	50	221	137	300	0.28
Soft	100	2	50	217	128	300	0.297
Soft	20	0.5	40	205	146	700	0.084
Soft	20	0.5	40	212	157	700	0.079
Soft	20	0.5	40	233	172	700	0.087

### Ex vivo temperature measurement

**Figure 3.**
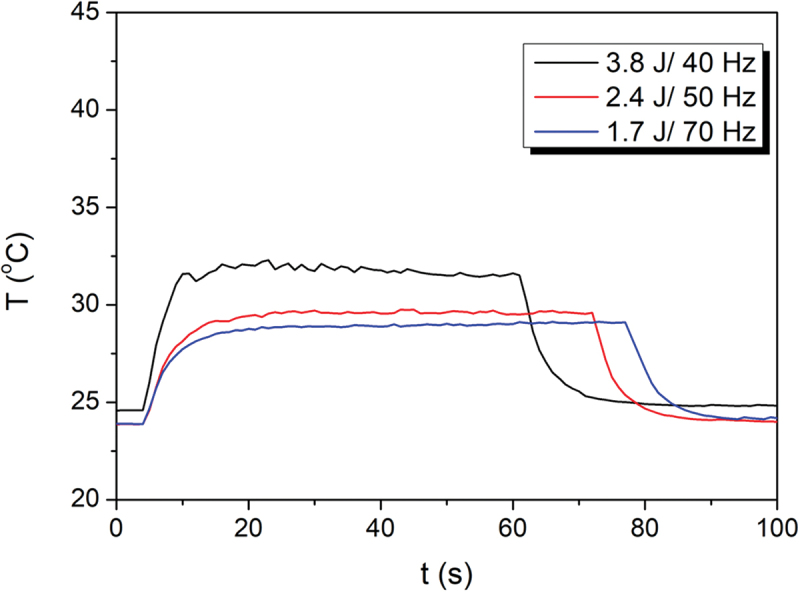
Temperature live tracking in different laser settings.

In all trials of the ex vivo experimental setting, an increase in the temperature during laser activation was noticed. When 152 W was used, 32°C was the highest temperature observed. In the trial of 120 W, a peak of 30°C at 42–43 s after the beginning of the procedure was detected. In the last trial of 105 W, the highest temperature at about 28°C was recorded after 41–42 s. In all trials, temperature plateau was observed. The results of temperature live tracking are presented in Figure 3.

T: temperature

t: time

### Clinical study

In total 35 patients, 34 males and one female patient, were included in the study. Patients’ and stones’ characteristics as well as perioperative outcomes are presented in Table 2. The mean age of the patients was 56 ± 14 years whereas the mean stone size was 35.5 ± 14.8 mm. All patients were stone-free following the procedure and the mean operative time was calculated to be 43 ± 18 min. The hospitalization duration was one day in all patients except for one who needed prolonged hospitalization for three days due to postoperative hematuria (Clavien-Dindo I). The complication was managed conservatively. Additional intra- or postoperative complications were not observed.

**Table 2.** Patients’ demographics, stone characteristics and perioperative outcomes.

Number of cases (n)	35
Age (years, mean±SD)	56 ± 14
Male gender (n (%))	34 (97.1)
BMI (kg/m^2^, mean±SD)	26.4 ± 5.0
Stone characteristics	
Total stone size (mm, mean±SD)	35.5 ± 14.8
Operative time (min, mean±SD)	43 ± 18
Number of stones (n (%))	
1	23 (65.7)
2	5 (14.3)
>2	7 (14)
Stone clearance (n (%))	35 (100)
One-day hospital stay (n (%))	34 (97.1)
Complications (n (%))	1 (2.9)
Prolonged hematuria treated	1 (2.9)

SD: Standard Deviation.

BMI: Body Mass Index.

## Discussion

In the present study, we evaluated the safety, efficacy and effectivity of HP laser lithotripsy in the treatment of BL. In vitro and ex vivo experimental settings along with clinical observation were performed. In vitro, HP settings were associated with significantly higher ARs regardless of the stone type. When evaluating the temperature patterns, the highest observed temperature was in the use of power setting of 152 W. It is well-documented in the literature that the threshold for tissue injury is 43°C [12]. In our experimental study with continuous irrigation flow, the temperature never reached the threshold of 43°C. The safety and efficacy of HP bladder lithotripsy were proven in the clinical investigation of 35 patients as well. With a mean operative time of 43 ± 18 min, all cases were stone-free after the procedure regardless of the stone size. Only one patient developed a minor complication and stayed at the hospital for more than one day.

Most published studies are clinical and utilize the Lumenis Ho:YAG laser system. In the systematic review of Becker and his colleagues, Ho:YAG laser seemed the most effective treatment option for urolithiasis. The authors focused on the ‘Moses’ mode of Lumenis Ho:YAG system and its advantages related to less retropulsion as it is considered the optimized energy transfer from the laser fiber to the stone [13]. Ballesta Martinez *et al*. published data on the stone AR in various power settings in Quanta Cyber: Ho 150 (Quanta System) [15]. Higher ARs were described in the HP settings regardless of the stone type. Besides, the time needed to reach the target energy was less when HP was used. The highest AR was recorded in the Virtual Basket mode with HP settings of the lasing system.

In our study, the Quanta Cyber: Ho 150 (Quanta System, Samarate, Italy) with 550 μm Quanta optical fiber (Quanta System, Samarate, Italy) was utilized in several power settings. Sea *et al*. performed an experimental study with different power settings in Ho:YAG lithotripsy [16]. In their study, 30 identical stone phantoms of calcium sulfate dehydrate were used. The researchers utilized a 365 μm optical fiber (Lumenis) and a 5 Fr ureteral catheter, and provided evidence that Ho:YAG lithotripsy at LP settings contributes to less retropulsion than HP energy which is also related to better fragmentation results. Black *et al*. showed that in short pulse mode, the low frequency and power (20 W) in a 2-mm distance between the laser tip and the stone was associated with more effective fragmentation [17]. Various in vitro and clinical studies focus on a new super-pulsed Thulium fiber laser which has shown encouraging results in the AR [18–20]. However, more experimental and clinical trials, especially with regard to HP settings, are deemed necessary and effectiveness and safety must be further evaluated.

Irrigation fluid temperature is an important parameter that should be considered particularly when using HP settings. Most of the published experimental studies evaluate temperature during HP Ho:YAG lithotripsy in ureteroscopic procedures. Aldoukhi *et al*. performed an in vivo experimental study in four female pigs undergoing retrograde ureteroscopy after the placement of thermocouples in their pelvic-calyceal system [10]. The authors concluded that HP settings (40 W) could increase the temperature inside the upper urinary tract in an injurious way (84.8°C, 63.9°C), especially with low irrigation flow. The safety of HP Ho:YAG lithotripsy was further investigated in the experimental study of Winship *et al* [14]. They used a flexible or semi-rigid ureteroscope through an 11/13Fr ureteral access sheath. The sheath was inserted into a bag containing 250 mL of normal saline. A thermocouple was placed next to a 365 μm fiber. Temperatures over the threshold of 43°C were recorded for 1 s at common irrigation pressures, especially with the flexible ureteroscope and power settings ≥ 10 W. The high temperature returned to a safe level within 5 s. The primary endpoints of their study were the right use of irrigation, the limited use of laser activation and the adequate time for temperature decrease.

Our experimental trial was conducted in the bladder of the porcine model in contrast to Aldhoukhi *et al*. in which the experimental trial was performed in the upper urinary tract. No similar to our ex vivo experimental study has been published in the literature. Based on our findings, the temperature even in the highest power settings did not approach 43°C, staying always below 32°C. The good irrigation flow can be explained by the larger diameter of the cystoscope and its working channel which provides better and faster fluid outflow. These measurements suggest that when the outflow channel is open, none of the power settings can have any negative impact on the bladder wall tissue. Noureldin *et al*. performed an in vivo experimental study in female pigs to measure the intrarenal temperature after using different laser settings, different ureteral access sheaths and different kind of irrigation (gravity or manual pump irrigation) [21]. Manual pump irrigation was related to safe temperatures regardless the laser settings and the size of the ureteral access sheath used. Consequently, good irrigation flow seems to be the key for keeping the temperatures low even with HP settings > 60 W. In addition to the temperature and the fluid irrigation, the anatomy of the gas bubble can compromise the safety of laser lithotripsy as a larger gas bubble is associated with higher applied pressure. Ho:YAG is characterized by increased lateral bubble expansion which may constitute a drawback regarding the safety of the procedure [22]. However, the increased capacity of the urinary bladder provides the capability to overpass this obstacle even for power > 100 W.

In the clinical part of the study, all patients treated with HP lithotripsy presented stone-free regardless of the initial stone size. Our results seem promising and are in line with the published literature. Bhat *et al*. conducted a prospective clinical study to compare LP to HP bladder lithotripsy utilizing Ho:YAG lithotripsy [9]. Ten patients were treated with LP settings and were compared to ten patients who underwent HP bladder lithotripsy. HP settings were related to decreased operative time without affecting intra-operative complication and stone-free rate. Consequently, the results were similar to that of the current study. The application of both Ho:YAG laser power settings in the treatment of BL was also evaluated under local anesthesia with comparable outcomes [23].

However, this study presents certain limitations. Firstly, in the experimental trials, the most important limitations were the use of two certain stone types (hard and soft) and the absence of AR comparison in different irrigation parameters. Besides, the ex vivo experimental set-up cannot replicate real clinical scenario. Several anatomical and functional alterations exist in clinical practice that cannot be predicted. Nonetheless, the baseline body temperature could also affect the irrigation fluid temperature. Despite the absence of the heatsink effect, the outcomes of ex vivo models can be considered reliable [24]. Although the design of the experimental settings implies an ideal testing environment with a reliable data collection scheme, more experimental studies are deemed necessary to compare the efficacy and safety of HP and LP bladder lithotripsy regarding the stone hardness and the AR. Finally, in our clinical study, potential limitations could be the relatively low number of included patients and the absence of a comparison group. Additionally, statistical analysis for the selection of the sample size was not performed. However, to our knowledge, this is the first study including a combined in vitro and ex vivo experimental as well as a clinical investigation of Ho:YAG laser bladder lithotripsy using HP settings > 100 W.

## Conclusion

Bladder lithotripsy with power settings up to 100 W is a fast and effective treatment for bladder stones. The temperature measurements and the absence of major complications imply that HP settings do not compromise the safety of the procedure.

## Data Availability

Data available on request from the corresponding author.

## List of Abbreviations

AR: Ablation Rate
BL: Bladder lithiasis
Ho:YAG: Holmium: Yttrium-Aluminum-Garnet
LP: Low-Power
HP: High-Power
